# Presence of *Anaplasma* spp. and Their Associated Antibodies in the Swedish Goat Population

**DOI:** 10.3390/ani13030333

**Published:** 2023-01-17

**Authors:** Sara Lysholm, Frida Ådén, Anna Aspán, Ann Högberg, Jonas Johansson Wensman, Anna Omazic

**Affiliations:** 1 Department of Clinical Sciences, Swedish University of Agricultural Sciences, 750 07 Uppsala, Sweden; 2 Animal and Human Health Program, International Livestock Research Institute, Nairobi 00100, Kenya; 3 Department of Microbiology, National Veterinary Institute, 751 89 Uppsala, Sweden; 4 Department of Chemistry, Environment and Feed Hygiene, National Veterinary Institute, 751 89 Uppsala, Sweden; 5 Department of Disease Control and Epidemiology, National Veterinary Institute, 751 89 Uppsala, Sweden

**Keywords:** Anaplasmosis, *Anaplasma* spp., *Anaplasma phagocytophilum*, *Anaplasma ovis*, *Anaplasma capra*, goat, *Ixodes ricinus*, ticks, tick-borne diseases

## Abstract

**Simple Summary:**

Anaplasmosis is a bacterial disease that has a severe impact on livestock production, such as reduced milk yield, poor growth and increased susceptibility to other diseases. This pilot study investigated the presence of *Anaplasma* bacteria and associated antibodies in blood samples collected from a subset of the Swedish goat population. The samples were analysed using polymerase chain reaction and an enzyme-linked immunosorbent assay. One of 40 goats tested positive for the presence of genetic material from the bacteria, while 33% of the serum samples contained antibodies for anaplasmosis. These results indicate that anaplasmosis is widespread in the goat population in certain areas of Sweden. More research is needed to understand the impact of anaplasmosis on Swedish goats and goat farmers.

**Abstract:**

Anaplasmosis is a tick-borne disease that has a severe impact on livestock production and welfare. The aim of this pilot study was to investigate the presence of *Anaplasma* spp. and associated antibodies in a subset of the Swedish goat population. In 2020, six goat herds located in different parts of Sweden were visited and whole blood and serum samples were collected. The whole blood samples (*n* = 40) were analysed for the presence of *Anaplasma phagocytophilum*, *A. ovis* and *A. capra* using quantitative and conventional polymerase chain reaction (PCR). The serum samples (*n* = 59) were analysed for the presence of antibodies to *Anaplasma* spp. using a commercial competitive enzyme-linked immunosorbent assay, and the same analysis was carried out on additional serum samples previously collected in 2018, 2019 and 2020 (*n* = 166). One goat (2.5%) tested positive for the presence of *A. phagocytophilum* genetic material, while the seropositivity rate ranged from 20 to 71%, depending on the surveyed year and area. These results indicate widespread exposure to *Anaplasma* spp. in the Swedish goat population. To inform future risk assessments and control efforts, further research is warranted to determine the prevalence of anaplasmosis and its impact on goat farming in Sweden.

## 1. Introduction

Anaplasmosis is caused by different species of the bacterial genus *Anaplasma* spp. and is a common disease in ruminants throughout the world [1]. In Sweden, earlier research on anaplasmosis has focused on cattle [2], sheep [3], horses [4], dogs [5], wild ruminants [6,7] and humans [8]. At present, the prevalence of *Anaplasma* spp. among Swedish goats is unknown. According to the Swedish Board of Agriculture, there are approximately 2400 goat farmers in Sweden, who together keep approximately 20,000 goats. More than 80% of these farmers keep small herds of between one and nine goats, less than 10% keep goats for dairy production and production of goat meat for sale is very rare. However, the number of goats and goat farmers has increased markedly in recent years, which necessitates greater knowledge about disease prevalence in Swedish goats [9].

*Anaplasma* spp. are Gram negative and obligate intracellular bacteria. They infect different blood cells depending on their species [1] and cause a variety of symptoms ranging from subclinical infections with mild fever [10,11] to high fever, anorexia, respiratory symptoms and a sudden decrease in milk yield [11,12]. Infection with *Anaplasma* spp. has also been linked to poor growth, abortion and reduced fertility [12]. While anaplasmosis in itself is rarely fatal, the bacteria can cause leukopenia and impaired neutrophil and lymphocyte function, rendering animals more susceptible to potentially deadly secondary infections [13]. Goats can be affected by a number of species, for instance *A*. *phagocytophilum*, *A*. *ovis* and *A*. *capra* [14,15,16]. Of these, both *A*. *phagocytophilum* [17] and *A*. *capra* [18,19] are zoonotic, i.e., they can be transmitted between animals and humans. *A*. *ovis* is considered less pathogenic than other species and generally only causes a subclinical infection with mild fever [10,20]. 

*Anaplasma* spp. Are transmitted by different tick species, for example *Ixodes* spp. and *Haemaphysalis* spp. In Sweden, three *Ixodes* spp. ticks are endemic and serve as vectors for *Anaplasma* spp., namely, *I. ricinus*, *I*. *persulcatus* and *Haemaphysalis punctata* [21,22,23,24,25]. *I*. *ricinus* is the most abundant tick species in Sweden, and in 2008 was estimated to be present throughout central and southern Sweden, as well as in large parts of northern Sweden [22]. Since the 1980s, the distribution of *I*. *ricinus* has increased considerably [21,23], which is believed to be due to a warming climate with mild winters and a longer vegetation season, an increase in the number and dispersion of the mammal host populations [22,23,24,26], and human activities such as land use changes and increased national and international travel [26]. As the climate continues to warm, it is likely that the number and geographical dispersion of ticks will continue to grow [22,23,24,27], which in turn will increase the risk of tick-borne diseases, such as anaplasmosis, in Swedish goats. Swedish legislation dictates that all goats should have free access to pasture for a stipulated number of months from 1 May to 15 October. The number of months varies depending on location in the country; the further north, the fewer months [28]. As a result, the goats can potentially be exposed to ticks and *Anaplasma* spp. during the period when the ticks are most active. At present, tick vectors for *A. phagocytophilum*, *A. ovis* and *A. capra* are all present in Sweden [29]. Therefore, the aim of this study was to investigate the presence of genetic material from *A. phagocytophilum*, *A. ovis* and *A. capra*, as well as antibodies to *Anaplasma* spp., in a subset of the Swedish goat population. 

## 2. Materials and Methods

### 2.1. Sample Collection

#### 2.1.1. Samples Collected in 2020

**Figure 1:**
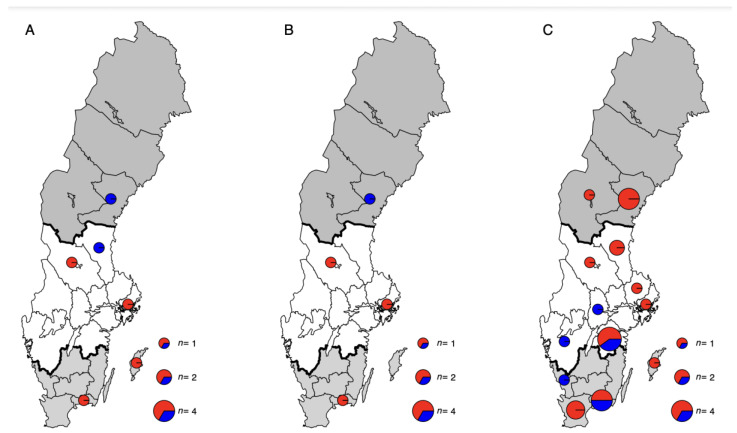
Maps of Sweden showing the locations; North (dark grey), Central (white) and South (light grey), of collected serum samples from goats for analysis of presence of *Anaplasma* spp. antibodies. The maps show the location and analysis results, positive herds (red) and negative farms (blue), of (**A**) samples collected in 2020, (**B**) samples collected 2019 and (**C**) samples collected from the CAE control programme.

The study was designed as a cross-sectional study. In total, six milk-producing and meat-producing goat farms in Sweden were contacted by e-mail and invited to participate in the study, and all agreed to take part. All the contacted farmers were members of the Swedish Goat Breeding Association and Swedish artisan dairy producers. They had been selected using convenience sampling based on their previous willingness to participate in research projects as well as their region of residence in Sweden, as the intention was to include farms in different parts of the country. The location of each herd is indicated as North, Central or South. North Sweden includes Jämtland, Norrbotten, Västerbotten and Västernorrland counties, Central Sweden comprises Dalarna, Gävleborg, Uppsala, Värmland, Västmanland, Stockholm, Södermanland, Örebro and Östergötland counties, and South Sweden consists of Blekinge, Gotland, Halland, Jönköping, Kalmar, Kronoberg, Skåne and Öland counties (Figure 1). The herd size varied considerably on the different farms, ranging from 10 to 99 goats, with an average of 52 animals. The herds were visited between August and September 2020, and on each farm blood samples were collected from nine to ten clinically healthy adult female goats aged one year or older. Information on acaricide use and whether the farmers regularly noticed ticks on their goats, was also obtained. For the herd that consisted of ten goats, all the animals were sampled, while in the larger herds, samples were obtained from every second or third goat in close proximity to the researchers. The blood samples were collected from the jugular vein using sterile needles, with whole blood collected in EDTA tubes (BD vacutainer, Plymouth, UK) and serum in vacutainer tubes without additives (BD vacutainer, Plymouth, UK). The samples were kept in a cool box during the day. At the end of each day, the EDTA whole blood samples were aliquoted into 1.5 mL Eppendorf tubes and frozen at −78 °C until analysis. The serum samples were centrifuged, whereby the serum was separated, transferred to 1.5 mL Eppendorf tubes, and placed in a −20 °C freezer until analysis. After one herd visit, it was not possible to centrifuge the serum samples within 24 h of sampling; therefore, the blood was left standing in a refrigerator overnight to allow the serum to separate, and the serum was then transferred to cryotubes the next morning. One of these samples did not coagulate and was therefore omitted from the study. 

#### 2.1.2. Samples Collected in 2019

Serum samples from a previous study conducted in 2019 were analysed for the presence of antibodies to *Anaplasma* spp. The study design has previously been described by Persson et al. [30]. In brief, the samples were collected from ten Swedish goat dairy farms that had been identified online either through their webpage or on social media. In each herd, serum samples were collected from the jugular vein from twenty or more lactating adult goats, and the selection of individuals to sample was made with the objective of obtaining as wide a variety of ages as possible. Of the 214 serum samples collected, fifty randomly selected samples from five herds were included in the current study. The herds were located in the northern and central parts of Sweden (Figure 1), and the above-mentioned geographical subdivision was also applied here. Two of the farms were visited in both 2019 and 2020, and one goat was sampled in both years.

#### 2.1.3. Samples Collected as Part of a Control Programme in 2018 and 2020

Serum samples from goats were acquired from 2018 and 2020 (*n* = 95 and *n* = 21, respectively) from Sweden’s National Veterinary Institute (SVA). The samples were collected by veterinarians and submitted to SVA as a part of a control programme for caprine arthritis encephalitis (CAE). These samples were obtained along with information on the geographical location of the sampled herds throughout Sweden (Figure 1). The same above-mentioned geographical subdivision system was used here. The samples had been stored at −70 °C until analysis. Unfortunately, no information on the sex, age or herd of origin, for example, was available for the analysed samples. 

### 2.2. cELISA

To determine the presence of antibodies to *Anaplasma* spp., a total of 225 serum samples were analysed using a commercial competitive enzyme-linked immunosorbent assay (cELISA; Veterinary Medical Research and Development, Pullman, WA, USA). According to the manufacturer, the cELISA detects antibodies to *A. marginale*, *A. ovis* and *A. centrale* in bovines. The sensitivity and specificity of the utilised assay has been estimated to be 96.5% and 98.1%, respectively, for the detection of antibodies to *A. ovis* in sheep [31]. The plates were utilized, validated and interpreted according to the instructions provided by the manufacturer and the results were categorised as positive or negative.

### 2.3. DNA Extraction and PCR

DNA was extracted from a total of 40 EDTA blood samples collected in 2020, randomly selecting six to seven samples from each herd. Using the DNeasy Blood and Tissue kit according to the manufacturer’s instructions (Qiagen, Wrocław, Poland), 100 µL of extracted DNA was obtained and then stored at −20 °C until further analyses.

To detect DNA from *A. phagocytophilum,* a quantitative PCR (qPCR) was used according to Henningsson et al. [32]. The primers and probe sequences are shown in Table 1 [32]. The positive control was acquired from SVA, and nuclease-free water was used as a negative control. The preparation of each individual sample consisted of 2 µL of the DNA sample, or a positive or negative control mixed with 6.25 µL of Taqman Fast virus 1-step Master Mix, 1.5 µL of each primer, 0.375 µL probe and 13.375 µL nuclease-free water. The following thermocycler (CFX96 Real-Time System, Bio Rad, Hercules, CA, USA) conditions were used: initial denaturation at 95 °C for 20 min, followed by 40 cycles of denaturation at 95 °C for 3 s and finally annealing and extension at 60 °C for 30 min.

For *A. ovis,* a conventional PCR was used. The primer sequences are shown in Table 1 [33]. The positive control came from Botswana and was eluted from Whatman FTA filter paper (Sigma-Aldrich, Saint-Louis, MO, USA), having previously tested positive for the presence of DNA from *A. ovis* [34]. As a negative control, nuclease-free water was used. The preparation of each individual sample consisted of 5 µL DNA, or a positive or negative control, mixed with 12.5 µL AmpliTaq Gold 360 MasterMix, 0.4 µmol of each primer and 5.5 µL nuclease-free water. The following conditions for the thermo-cycler (2720 Thermal cycler, Applied Biosystem, Waltham, MA, USA) were used: initial denaturation at 95 °C for 10 min followed by 40 cycles of denaturation at 95 °C for 30 s, annealing at 50 °C for 60 s and extension at 72 °C for 60 s, followed by a final extension at 72 °C for 7 min. The PCR products were then analysed by gel electrophoresis in a 1% agarose gel and visualised under UV light. The expected length of the PCR product was 92 base pairs (bp).

For *A. capra,* a conventional PCR was used. The primer sequences are shown in Table 1 [14]. The Master Mix consisted of 12.5 µL AmpliTaq Gold 360 MasterMix, 1 µL of each primer and 8 µL nuclease-free water, then mixed with either 2.5 µL DNA or 2.5 µL positive control sample (see description below) or 2.5 µL nuclease-free water used as the negative control, summing up to a total volume of 25 µL. The following conditions for the thermocycler (Bio-Rad S1000^TM^ Thermal Cycler) were used: initial denaturation at 95 °C for 10 min followed by 35 cycles of denaturation at 95 °C for 30 s, annealing at 55 °C for 30 s and extension at 72 °C for 60 s, followed by a final extension at 72 °C for 7 min. PCR products were then analysed by gel electrophoresis in a 1% agarose gel and visualised under UV light. 

As a positive control sample for the *A. capra* analysis, DNA extracted from sheep blood previously diagnosed as positive for *A. capra* was used [3]. The positive whole blood sample was collected in 2007 from a diseased sheep on the island of Fårö, Sweden. The sample tested positive for *A. phagocytophilum* by qPCR and was therefore sent for DNA Sanger sequencing, whereby it was found that the dominant *Anaplasma* species in the sample was not *A. phagocytophilum*. Upon blasting the groEL sequence (1424 bp), a 99.9% similarity to deposited sequences of *A. capra* (GenBank accessed 09152021) was identified. When analysing extracted DNA from this sample with PCR [14], a single band of the expected size was produced. Thus, this DNA was used as positive control in the screening of the samples in the current study for the presence of *A*. *capra*.

### 2.4. Statistical Analysis

Data from the diagnostic analyses were compiled in Microsoft Excel (version 16.43, Microsoft, Washington, DC, USA). A confidence interval (CI) of 95% was calculated for cELISA and PCR results using the Minitab Express statistical software (version 19.2020.1.0, Microsoft, Washington, DC, USA). Proportions were compared using Fischer’s exact test and univariable logistic regression in Stata IC 16/1 (StataCorp LLC, College Station, TX, USA). Only univariable analysis was performed. Results were considered statistically significant if *p* < 0.05. 

### 2.5. Ethical Approval

Informed oral consent was obtained from the farmers prior to sample collection. They were informed that the data would be published online, but that the information about them would remain anonymous and confidential. The study received ethical approval from the Swedish Ethical Committee on Animal Research, (approval number C 148/13 and 5.8.18-15533/2018).

## 3. Results

### 3.1. Seropositivity Rates of Antibodies to Anaplasma spp. and Usage of Acaricides in 2020

**Figure 2:**
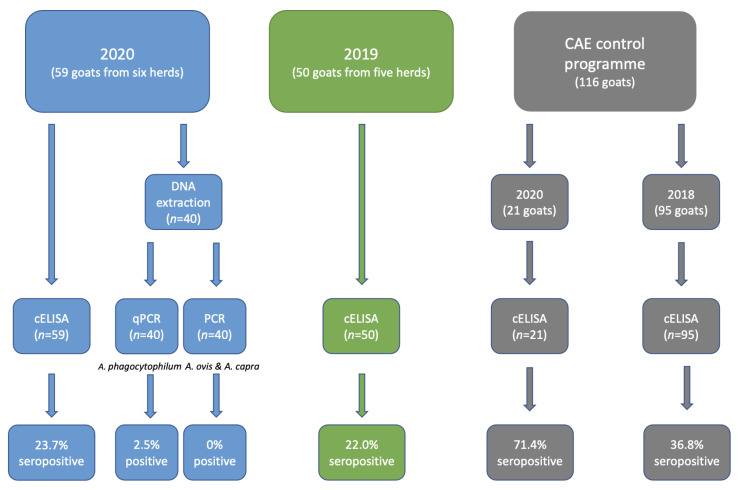
Illustration of the number of samples collected from goats in Sweden in 2020 and 2019, and in the CAE control programme, as well as the number of samples analysed for the presence of *Anaplasma* spp. and its specific antibodies using cELISA and PCR. The number of positive samples is also included. qPCR denotes quantitative/real-time PCR used to detect DNA from *A. phagocytophilum*, while PCR denotes conventional PCR used to detect DNA from *A. ovis* and *A. capra*.

In total, 225 serum samples were analysed for the presence of antibodies to *Anaplasma* spp. Of the samples collected in 2020 (*n* = 59), the seropositivity rate was 23.7% (Table 2; Figure 2). The proportion of seropositive goats was significantly higher in South Sweden (50%) than in Central Sweden (13.8%) (OR 6.25, 95% CI 1.59–24.6, *p* = 0.009), while no goats sampled in North Sweden were seropositive. The seropositivity rate in herd 6 (80%) was significantly higher than in the other seropositive herds (OR 16.0, 95% CI 1.79–143.2, *p* = 0.013). Information on whether the farmers had observed ticks on their goats and on acaricide usage was available from all herds except for herd 6. In three out of five goat herds (herds 1, 2 and 5), the farmers regularly observed ticks on their goats in summer. Acaracides were only used in herd 2, where a pour-on solution containing cypermethrin was applied to male goats at the start of the grazing season each year. 

Of the samples collected in 2019, the seropositivity rate was 22.0% (Table 2; Figure 2). No statistically significant difference between North Sweden (13.3%) and Central Sweden (35.0%) was observed (*p* = 0.090). The seropositivity rate in herd E (60%) was significantly higher than that in the other seropositive herds (OR 13.5, 95% CI 1.20–152.2, *p* = 0.035).

No statistically significant difference was found between the seropositivity rates in 2020 and 2019 (*p* = 1.000). Two herds were visited and sampled in both 2019 and 2020, and while both were seropositive in 2019 (10% and 20%, respectively), they were seronegative in 2020. One individual was sampled in both 2019 and 2020 and tested negative in both years. 

Among the samples acquired from SVA from 2020 and 2018, 15 and 35, respectively, were positive, resulting in seropositivity rates of 71.4% and 36.8%, respectively (Table 3; Figure 2). The seropositivity rate was significantly higher in 2020 than in 2018 (OR 4.29, 95% CI 1.52–12.1, *p* = 0.006). In both 2020 (OR 13.0, 95% CI 1.36–124.3, *p* = 0.026) and 2018 (OR 5.31, 95% CI 2.04–13.8, *p* = 0.001), the seropositivity rate was significantly higher in South Sweden than in Central Sweden. 

### 3.2. Presence of DNA from Anaplasma spp.

The samples (*n* = 40) analysed by qPCR showed one sample that was positive for *A. phagocytophilum,* resulting in a prevalence of 2.5%. This positive sample was collected from one of the herds located in South Sweden. All the samples were negative for *A. ovis* and *A. capra* (Figure 2). 

## 4. Discussion

*Anaplasma* spp. are tick-borne pathogens that can adversely affect livestock production and welfare. Furthermore, as certain species are zoonotic, the pathogens can also have a negative impact on human health. The present study analysed samples collected in 2020, 2019 and 2018 from different regions of Sweden as part of research studies and a disease control programme. To the authors’ knowledge, this is the first study that has detected genetic material from *A. phagocytophilum*, as well as *Anaplasma* spp. specific antibodies, in Swedish goats. 

In the present study, a cELISA was used to detect antibodies to *Anaplasma* spp. The cELISA is specific for an epitope of the major surface protein-5 (MSP-5) that is conserved among *Anaplasma* species [35] and, according to the manufacturer, detects antibodies for *A. marginale*, *A. ovis* and *A. centrale*. Of these, *A. marginale, A. ovis* and *A. centrale* are generally detected in tropical and subtropical areas [20,36] and have therefore never been found in Sweden. Furthermore, only some of their respective tick vector species are present in the country. The cELISA has been validated for the detection of *A. ovis* antibodies in sheep [31] and has been used on previous occasions to detect *A. phagocytophilum* antibodies in sheep and cattle for example [37,38]. However, in a recent study, questions were raised about the assay’s suitability for the detection of antibodies to *A. phagocytophilum* in goats [39] as the authors stated that they had found high inter-run variability, although corresponding data were not presented in the article. To the authors’ knowledge, the assay has not been validated for detection of antibodies to *A. capra* and this should be borne in mind when interpreting the serological results of the present study. 

In 2020, the detected seropositivity rate was 23.7%, which is similar to that found in lambs in two regions of southern Sweden in a previous study [3]. However, it is considerably lower than results from previous studies on moose [7] and roe deer [6] in southern and central Sweden, where the detected seroprevalence was 100% and 85%, respectively. A potential reason for this variation is differences in bacterial ecotypes between wild and domestic ruminants [40]. The present results are also lower than levels observed in lambs in Norway, where a 60% seroprevalence was detected [41]. However, in all the previous studies in Sweden and Norway, samples were analysed with an indirect immunofluorescence antibody assay, a diagnostic method that is no longer available for analysis of *Anaplasma* spp. at SVA in Sweden. Therefore, comparisons between this study and previous studies should be made with caution. The seropositivity rate was significantly higher in South Sweden than in Central Sweden, and none of the goats sampled in North Sweden were seropositive. A plausible reason for this finding is that the climatic conditions in South Sweden are generally more favourable for ticks, with the region’s shorter winters and higher ambient temperatures [22]. In one of the six herds visited in 2020, a seropositivity rate of 80% was detected, which was significantly higher than the rate in the other herds studied. This herd was located on the island of Gotland, and in a previous study by Grandi et al. [3], lambs on Gotland were found not only to be more likely to be exposed to multiple species of *Anaplasma* spp., compared with lambs residing in mainland Sweden [3], but also at higher risk of being co-infected by them. 

Of the herds sampled in 2020, herds 3 and 4 reported that they had not observed ticks on their goats. In herd 3, none of the tested goats had antibodies for *Anaplasma* spp. This farm is located in the northern part of Sweden where fewer ticks generally are found compared to in the central and southern parts of the country [21]. In herd 4, the seropositivity rate was 20%, indicating that the goats were in fact exposed to ticks, in spite of the farmers not noticing it. Acaricides, namely cypermethrin in a pour-on solution, was only used in one of the herds (herd 3), but the owners of this herd only applied the solution on adult male goats. This indicates a need to educate goat farmers about the value of acaricide usage. Unfortunately, no information was available on acaricide use in herd 6, where an 80% seropositivity rate for *Anaplasma* spp. was observed. 

In 2019, the seropositivity rate was 22.0%, which was not significantly different from the level in 2020. All the surveyed herds were found in either Central or North Sweden, and the seropositivity rate was not significantly different between the two. The proportion of seropositive goats (60%) in one herd in Central Sweden was significantly higher than in the other seropositive herds. This herd was located close to Stockholm, potentially indicating a high prevalence of *Anaplasma* spp. and/or the tick vectors in this part of the country. Two herds were visited and sampled in both 2019 and 2020, and interestingly, while both herds were seropositive in 2019, they were both seronegative in 2020. While it seems counterintuitive for a herd to go from being seropositive to seronegative in the course of just one year, the change is likely to be due to different animals being sampled in 2019 and 2020, although one goat sampled in 2019 and 2020 tested negative in both years. It should also be noted that the number of seropositive animals in these herds was modest in 2019, with 10% and 20% of each herd testing seropositive. 

The detected seropositivity rates from samples submitted to SVA from herds in Central and South Sweden in 2020 and 2018 (as a part of the CAE control programme) were 71.4% and 36.8%, respectively. The seropositivity rate in South Sweden compared with Central Sweden was significantly higher in both 2018 and 2020. Potential explanations for this could be the higher prevalence of *Anaplasma* spp. and/or greater abundance of ticks in South Sweden than in central parts of the country. Furthermore, the seropositivity rate was significantly higher in 2020 than in 2018, but as the number of samples differed considerably between the years and as the study’s authors did not have access to important information on the sampled animals, for example with regards to the presence of clinical signs of disease at the time of sampling, this finding should be interpreted with caution. 

In previous studies, samples from various species have tested positive for *Anaplasma* spp. in conventional or real-time PCR analysis [2,3,7]. In the present study, one out of forty (2.5%) whole blood samples collected in 2020 tested positive for the presence of *A. phagocytophilum*. The positive animal was also seropositive for antibodies to *Anaplasma* spp., and originated from the island of Gotland, an area of Sweden previously shown to have *A. phagocytophilum* in sheep [3]. The presence of *Anaplasma* spp. genetic material in this study was considerably lower than results from previous studies on lambs and cattle in Sweden, where 20.0% [3] and 23.9% [2], respectively, tested positive for the presence of genetic material from *A. phagocytophilum*. However, the studies were conducted in different areas of Sweden and in different years, which can have an impact on tick abundance and activity for example. 

None of the samples in the current study tested positive for genetic material from *A. ovis* and *A. capra*, although the latter has been found on the island of Gotland [3]. The absence of animals that tested positive for *A. ovis* was expected, since tick vectors for the bacterium are not currently found in Sweden. The bacterium is detected more in subtropical and tropical areas [20]. 

Despite the interesting results generated by this study, there are some limitations. Even though the samples were collected and analysed from three separate years, the number of samples (*n* = 225) is comparatively small. Furthermore, the study design in both 2020 and 2019 was convenience-based, and none of the analysed samples, including those obtained from the CAE control programme, were randomly collected. As a result, the detected seropositivity rates need to be interpreted with caution. Moreover, while the authors are aware that the same two herds were visited in both 2019 and 2020 and that one animal was sampled in both years, there is no information about whether any of the visited herds were part of the CAE control programme. Therefore, it is possible that samples from the same individual were analysed more than once. Furthermore, for the samples that were collected in 2019 and those obtained from SVA, information regarding the health status of the sampled animals was not available. Lastly, the samples collected in 2020 and 2019 were from adult animals, while for the samples from SVA, no information on age was available. Access to this information would have enabled more detailed conclusions to be drawn from the study’s findings.

## 5. Conclusions

This study is the first to demonstrate the presence of *A. phagocytophilum* genetic material and antibodies for *Anaplasma* spp. in Swedish goats. One goat (2.5%) tested positive for the presence of genetic material from *A. phagocytophilum*, while the seropositivity rate in 2020, 2019 and 2018 ranged from 22 to 71%. Usage of acaricides to prevent tick exposure was low in the herds visited in 2020, which indicates a need to educate Swedish goat farmers about the value of acaricide treatment. The findings indicate that anaplasmosis is widespread in goats throughout the country, especially in South and Central Sweden. However, more extensive studies are warranted to map the presence of anaplasmosis accurately and elucidate the impact of the disease on goat production in Sweden. 

## Figures and Tables

**Table 1:** Sequences of the primers and probes used to detect DNA from *Anaplasma phagocytophilum*, *A. ovis* and *A. capra*. DNA from *A. phagocytophilum* was detected with quantitative PCR, while DNA from *A. ovis* and *A. capra* was detected using conventional PCR.

Pathogen	Primer Name	Sequence	Reference
*A. phagocytophilum*	Anaplasma-F	5′-TTTTGGGCGCTGAATACGAT-3′	[32]
*A. phagocytophilum*	Anaplasma-R	5′-TCTCGAGGGAATGATCTAATAACGT-3	
*A. phagocytophilum*	Anaplasma-probe	5′-FAM-TGCCTGAACAAGTTATG-3′	
*A. ovis*	An_ov_msp4_F	5′-TCATTCGACATGCGTGAGTCA-3′	[33]
*A. ovis*	An_ov_msp4_R	5′-TTTGCTGGCGCACTCACATC-3′	
*A. capra*	An_capr_groEL_F	5′-TGAAGAGCATCAAACCCGAAG -3′	[14]
*A. capra*	An_capr_groEL_R	5′-CTGCTCGTGATGCTATCGG -3′	

**Table 2:** Number of positive goats from herds in South, Central and North Sweden sampled in August–September 2020 and in 2019, including geographical location and their seropositivity rates.

Year	Herd	Geographic Location in Sweden	Number of Positive Samples (Number of Analysed Samples)	% Positive Samples (95% CI)
2020	1	Central	2 (9)	22.2 (2.81–60.0)
	2	Central	2 (10)	20.0 (2.52–55.6)
	3	North	0 (10)	0.0 (0–30.8) *
	4	South	2 (10)	20.0 (2.52–55.6)
	5	Central	0 (10)	0.0 (0–30.8) *
	6	South	8 (10)	80.0 (44.4–97.5)
	Total		14 (59)	23.7 (13.6–36.6)
2019 *	A	North	1 (10)	10.0 (0.25–44.5)
	B	North	3 (10)	30.0 (6.67–65.2)
	C	North	0 (10)	0.0 (0–30.8) *
	D	Central	0 (10)	0.0 (0–30.8) *
	E	Central	6 (10)	60.0 (26.2–87.8)
	Total		11 (50)	22.0 (10.0–33.7)

* One-sided 97.5% confidence interval.

**Table 3:** Number of positive samples from 2018 and 2020 collected in South and Central Sweden and unknown counties, including geographical location and seropositivity rates.

Year	Geographic Location in Sweden	Number of Positive Samples (Number of Analysed Samples)	% Positive Samples (95% CI)
2018	Central	14 (56)	25.0 (14.4–38.4)
	South	19 (30)	63.3 (43.9–80.1)
	Unknown	2 (9)	22.2 (2.81–60.0)
	Total	35 (95)	36.8 (27.2–47.4)
2020	Central	2 (6)	33.3 (4.33–77.7)
	South	13 (15)	86.7 (59.5–98.3)
	Total	15 (21)	71.4 (47.8–88.7)

## Data Availability

The data presented in this study are available on request from the corresponding author.
